# Effect of Quercetin Treatment on Mitochondrial Biogenesis and Exercise-Induced AMP-Activated Protein Kinase Activation in Rat Skeletal Muscle

**DOI:** 10.3390/nu12030729

**Published:** 2020-03-10

**Authors:** Keiichi Koshinaka, Asuka Honda, Hiroyuki Masuda, Akiko Sato

**Affiliations:** 1 Department of Health and Sports, Niigata University of Health and Welfare, 1398 Shimami-cho, Kita-ku, Niigata 950-3198, Japan; wtm19009@nuhw.ac.jp (A.H.); akiko-sato@nuhw.ac.jp (A.S.); 2 Department of Health and Nutrition, Niigata University of Health and Welfare, 1398 Shimami-cho, Kita-ku, Niigata 950-3198, Japan; masuda@nuhw.ac.jp

**Keywords:** AMPK, rat, skeletal muscle, quercetin, exercise

## Abstract

The purpose of this study was to evaluate the effect of chronic quercetin treatment on mitochondrial biogenesis, endurance exercise performance and activation levels of AMP-activated protein kinase (AMPK) in rat skeletal muscle. Rats were assigned to a control or quercetin group and were fed for 7 days. Rats treated with quercetin showed no changes in the protein levels of citrate synthase or cytochrome C oxidase IV or those of sirtuin 1, peroxisome proliferator-activated receptor gamma coactivator-1α or phosphorylated AMPK. After endurance swimming exercise, quercetin-treated rats demonstrated no differences in blood and muscle lactate levels or glycogen utilization speed compared to control rats. These results indicate that quercetin treatment does not stimulate mitochondrial biogenesis in skeletal muscle and does not influence metabolism in a way that might enhance endurance exercise capacity. On the other hand, the AMPK phosphorylation level immediately after exercise was significantly lower in quercetin-treated muscles, suggesting that quercetin treatment might provide a disadvantage to muscle adaptation when administered with exercise training. The molecular results of this study indicate that quercetin treatment may not be advantageous for improving endurance exercise performance, at least after high-dose and short-term therapy.

## 1. Introduction

Quercetin is one of the most common flavonoids found in many foods, including onions, red wines, blueberries and leeks [1]. Quercetin is a powerful antioxidant and has been shown to have cardioprotective and anti-inflammatory properties and to reduce the risk of metabolic syndrome and muscle atrophy [2,3,4,5]. In addition to its pharmacological effects, quercetin is thought to have possible ergogenic effects, especially in endurance exercise. Several human and rodent studies [6,7,8,9] demonstrated that quercetin treatment increased VO_2_ max and time to fatigue and improved performance in the 30-km time trial and maximal 12-min test.

It is well known that mitochondrial biogenesis in skeletal muscle plays a substantial role in determining endurance exercise performance. Exercise training improves performance and increases mitochondrial content. From this perspective, a landmark study conducted by Davis et al. [7] has received the most attention in the field of quercetin-related research. The authors demonstrated that relatively short-term (7 days) quercetin supplementation increased mitochondrial biogenesis in mouse skeletal muscle in the absence of exercise training and resulted in a remarkable improvement in endurance exercise capacity. However, a subsequent study in humans did not replicate this exercise-like effect on mitochondrial biogenesis [9], while another human study found only a weak effect [10]. Although the exact cause of this discrepancy is unclear, authors of previous studies hypothesized that quercetin’s effect on mitochondrial biogenesis may differ between human and rodents [10,11,12]. If this is true, it will have a major impact because rodent-derived cells and tissues are extremely beneficial for identifying bioactive substances that stimulate mitochondrial biogenesis and for analyzing the molecular mechanisms of plant-derived substances such as polyphenols. Thus far there is very little information regarding quercetin’s effect on mitochondria in skeletal muscle, despite the experimental importance of this issue. We hypothesized that quercetin treatment might not be a stimulus for inducing mitochondrial biogenesis even in rodent skeletal muscle.

Mitochondrial biogenesis is a complicated process in which peroxisome proliferator-activated receptor gamma coactivator (PGC)-1α has been shown to play a major role [13]. Increased activity of PGC-1α induced by PGC-1α deacetylation and phosphorylation results in stimulation of mitochondrial DNA (mtDNA) replication and gene expression. It is well documented that AMP-activated protein kinase (AMPK) plays a role in both deacetylation and phosphorylation [13]. AMPK activation increases PGC-1α deacetylase sirtuin (SIRT) 1 activity via nicotinamide adenine dinucleotide stimulation and also increases PGC-1α phosphorylation as a direct effect of AMPK kinase activity. Furthermore, AMPK activation increases both PGC-1α and SIRT1 protein expression levels, contributing to the activities of these molecules. In this context, AMPK is considered to be a master switch that induces mitochondrial biogenesis and it is expected to be activated by quercetin treatment if quercetin has an impact on mitochondria. Indeed, in cultured L6 [14] and C2C12 cells [15,16], AMPK activation levels were successfully increased after incubation with quercetin. However, to the best of our knowledge, no studies thus far have examined the in vivo effects of acute and chronic oral quercetin treatment on muscle AMPK activation levels. Importantly, a previous study reported that resveratrol, another polyphenol that promotes mitochondrial biogenesis, stimulated muscle AMPK and increased mitochondrial biogenesis only in cultured cells and not following in vivo treatment [17]. Therefore, we consider it important to identify quercetin’s effect on muscle AMPK in vivo to determine whether quercetin impacts mitochondrial biogenesis in living organisms.

In contrast to the findings discussed above, accumulating studies have suggested that antioxidant supplementation hampers several acute exercise-induced cell signaling responses as well as training-induced adaptations, including mitochondrial biogenesis [18,19]. Since quercetin is a powerful antioxidant, when administered along with exercise it might have a detrimental effect on exercised skeletal muscle. Several studies have demonstrated that exercise stimulates AMPK activation [20,21]. Therefore, we hypothesized that quercetin treatment would result in reduced AMPK activation in response to exercise. In the present study, in addition to focusing on the effects of quercetin treatment on the basal (resting) level of AMPK in skeletal muscle, we also investigated the impact of quercetin on exercise-induced AMPK activation.

The primary purpose of this study was to evaluate the effect of chronic quercetin treatment on mitochondrial biogenesis in rat skeletal muscle and to compare our results with those of the previous landmark study [7]. Quercetin’s possible ergogenic effect was also assessed by measuring the rate of glycogen expenditure during submaximal endurance exercise. The second goal of this study was to investigate the effect of chronic quercetin treatment on AMPK activation both at baseline and immediately after acute exercise. To further characterize the possible effect of quercetin on skeletal muscle, AMPK activation levels were measured both after acute administration of quercetin in vivo and acute treatment in vitro.

## 2. Materials and Methods 

### 2.1. Animals

This research was approved by the Animal Studies Committee of Niigata University of Health and Welfare (29015-01013). Male Wistar rats (4 weeks, *n* = 65) were obtained from CLEA Japan (Tokyo, Japan). Rats were housed individually at constant room temperature (23 °C ± 1) in a 12-h light (06:00−18:00 h)/12-h dark cycle and were provided standard laboratory chow (MF: Oriental Yeast, Tokyo, Japan) and water ad libitum till experimental day (1 week later).

### 2.2. Quercetin Stimulation in Vitro

After 3-h fasting (09:00), rats were anesthetized by pentobarbital sodium (50 mg/body weight (BW)), and epitrochlearis muscles, located in the upper arm, were dissected out. The muscles were then incubated with shaking for 30 min at 35 °C in 4 mL of oxygenated Krebs-Henseleit buffer containing 8 mM glucose, 32 mM mannitol and 0.1% radioimmunoassay-grade bovine serum albumin, in the absence or presence of 100 μM quercetin (ChromaDex, Los Angeles, CA, USA), 100 μM resveratrol (Fujifilm Wako Pure Chemical, Osaka, Japan) or 0.5 mM 5-aminoimidazole-4-carboxamide-1-beta-d-ribofuranoside (AICAR) (Tronto Research Chemical, North York, Canada) in 0.1% DMSO. Flasks were gassed continuously with 95% O_2_/5% CO_2_ during incubation. After incubation, muscles were clamp-frozen in liquid nitrogen for western blot analysis.

### 2.3. Acute Effect of Quercetin Administration

After 3-h fasting, rats were orally administrated either orange-flavored Tang (Kraft Foods, Northfield, IL, USA) [7] as control or quercetin (25 mg/kg BW) mixed with Tang. Then, rats were maintained in a fasting and resting condition for 4 h. After that, epitrochlearis muscles were dissected out under anesthesia and clamp-frozen in liquid nitrogen for western blot analysis. 

### 2.4. Chronic (7 Days) Effect of Quercetin Treatment

Rats were randomly assigned to either a control or quercetin group and were maintained under ad libitum feeding for 7 days. During this period, rats were given once-daily oral supplementation of either Tang or quercetin as described above, with daily measurement of food intake. On day 7, the last supplementation was administered at 17:00. On the next morning (06:00), food was removed and rats were maintained in the fasting state for 3 h. After that, soleus and epitrochlearis muscles from some rats were dissected out under anesthesia and clamp-frozen in liquid nitrogen for western blot analysis and evaluation of muscle metabolites. Also, epididymal and retroperitoneal fat pads were removed and weighed. The remaining rats were used for endurance exercise testing before sacrifice.

### 2.5. Endurance Exercise Testing

On subsequent 2 days before testing, all rats have become accustomed to swimming for 10 min/day without a weight. On the testing day, after 3-h fasting, rats performed a swimming exercise for 1 h with a weight equal to ~2.5% of body weight. Immediately after exercise, blood was taken by tail-tip cut and then rats were rapidly anesthetized with isoflurane. Epitrochlearis muscles were dissected out under anesthesia and clamp-frozen in liquid nitrogen for measurement of muscle metabolites and western blot analysis.

### 2.6. Blood Parameters

Blood glucose and lactic acid levels were measured using product Glutest Every (Sanwa Kagaku, Nagoya, Japan) and Lactate pro (Arkray, Kyoto, Japan), respectively.

### 2.7. Western Blot Analysis

Epitrochlearis and soleus muscles were homogenized as previously described [21]. Primary antibodies were purchased as follows. From Cell Signaling Technology (Beverly, MA, USA); anti-AMPK-α, phospho-(p-)AMPK (Thr172), citrate synthase (CS), cytochrome C oxidase (COX) IV and SIRT1. From Merck (Temecula, CA, USA); anti-PGC-1α, monocarboxylate transporter (MCT) 1, MCT4 and MCT4. Anti-carnitine palmitoyltransferase (CPT) 1B, glucose transporter (GLUT) 4 and glyceraldehyde-3-phosphate dehydrogenase (GAPDH) were obtained from Abcam (Tokyo, Japan), Biogenesis (Poole, United Kingdom) and Sigma-Aldrich (St. Louis, MO, USA), respectively. Equal protein concentrations were loaded in each lane and also visually confirmed by coomassie brilliant blue staining of the blot membrane. The phosphorylated AMPK abundance was normalized by AMPK-α abundance and other proteins were normalized by GAPDH abundance.

### 2.8. Muscle Metabolites

Epitrochlearis muscles were homogenized in 0.3 M perchloric acid (PCA) and the extracts were used to measure glycogen by the amyloglucosidase method [22]. The remaining PCA extracts were centrifuged at 1000 *g* for 10 min at 4 °C. After centrifugation, the supernatant was collected and neutralized by the addition of 2M KOH, followed by fluorometric measurements of lactate [23].

### 2.9. Statistics

Values are expressed as means ± SE. Differences between groups were determined using an unpaired *t*-test. *p* < 0.05 was considered significant.

## 3. Results

### 3.1. Quercetin Stimulation In Vitro

**Figure 1:**
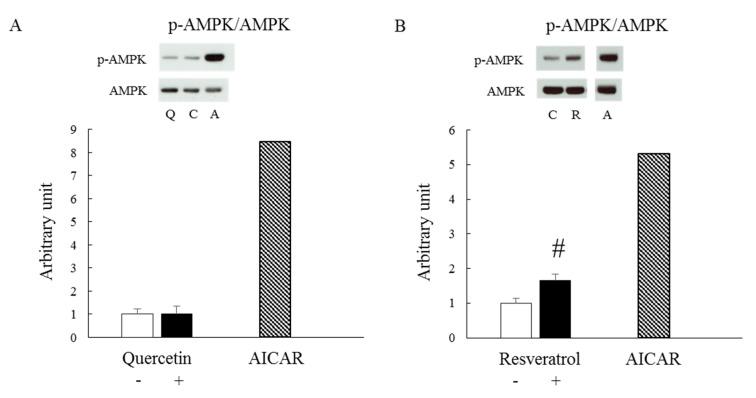
Effect of quercetin stimulation on AMPK phosphorylation in vitro. Isolated epitrochlearis muscles were incubated for 30 min either in the absence (-) or presence (+) of 100 μM quercetin (**A**) or 100 μM resveratrol (**B**). Values are means ± SE (*n* = 4–5). Sample stimulated with 0.5 mM 5-aminoimidazole-4-carboxamide-1-beta-d-ribofuranoside (AICAR) was used as a positive control (*n* = 1). A representative blot is shown above each figures. The obtained values of p-AMPK were normalized with the value for AMPK. #*p* < 0.05 vs. resveratrol (-). C: control (absence of stimulant), Q: quercetin, R: resveratrol, A: AICAR.

We first examined whether quercetin affected AMPK in mature, intact skeletal muscle by measuring AMPK phosphorylation at Thr 172, a critical site for AMPK activation (Figure 1A). Following the short, 30-min incubation period with epitrochlearis muscle, AICAR, a pharmacological AMPK agonist, successfully increased the AMPK phosphorylation level, whereas quercetin did not. In contrast, resveratrol, which is another polyphenol that is known to be an AMPK stimulant [17,24], increased AMPK phosphorylation levels after incubation for the same duration (Figure 1B).

### 3.2. Acute Effect of Quercetin Administration

**Figure 2:**
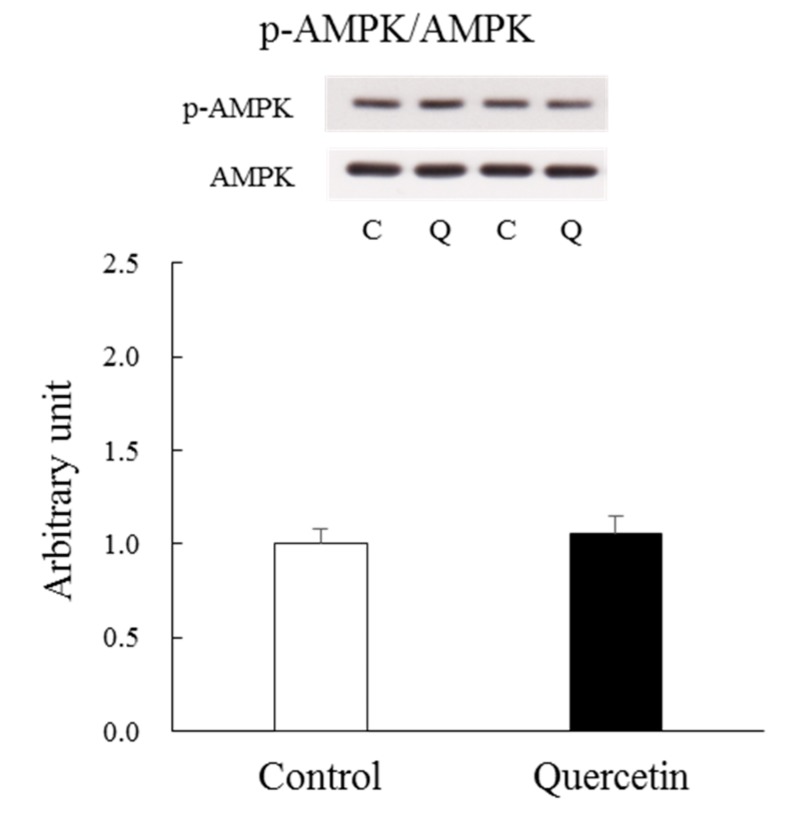
Acute effect of quercetin administration on AMPK phosphorylation. Epitrochlearis muscles were dissected out 6 h after oral quercetin or vehicle (control) administration. A representative blot is shown above figure. The obtained values of p-AMPK were normalized with the value for AMPK. Values are means ± SE (*n* = 6). C: control, Q: quercetin.

No previous studies have examined the effect of a single quercetin application to AMPK in skeletal muscle in vivo. A positive effect on AMPK would suggest that quercetin facilitates mitochondrial biogenesis. However, we found that AMPK phosphorylation levels were unaffected by a single quercetin application to the epitrochlearis muscle (Figure 2).

### 3.3. Chronic (7 days) Effect of Quercetin Treatment (with Epitrochlearis Muscle)

**Figure 3:**
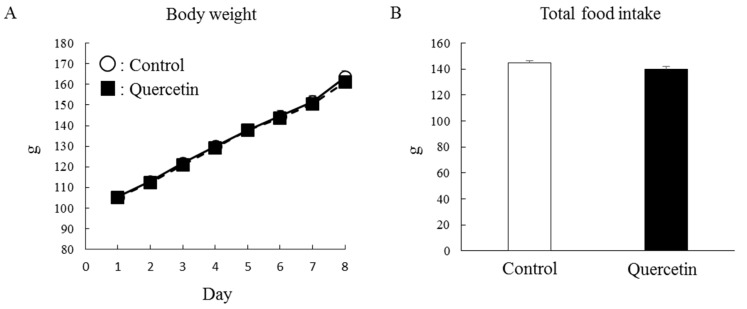
Effects of 7-day quercetin treatment on body weight and food intake. Rats were assigned to either a control or quercetin groups and were maintained under ad libitum feeding for 7 days. During this period, daily body weight (**A**) and total food intake (**B**) were measured. Values are means ± SE (*n* = 13–14).

**Figure 4:**
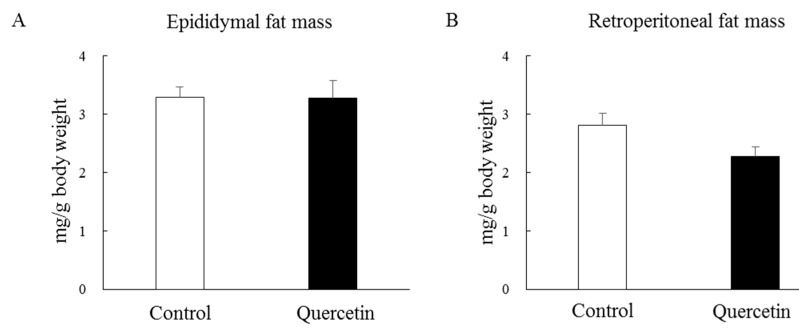
Effects of 7-day quercetin treatment on fat-pad mass. Rats were assigned to either a control or quercetin groups and were maintained under ad libitum feeding for 7 days. After that, epididymal (**A**) and retroperitoneal (**B**) fat-pads were removed and weighed. Values are means ± SE (*n* = 6–7).

To investigate whether repeated quercetin administration affected mitochondrial biogenesis, rats were supplemented with quercetin for 7 days. Body weight increased gradually during the treatment period but the rate of body weight gain was identical between the control and quercetin groups (Figure 3A). The rats in both groups ate the same amount of food throughout the treatment period (Figure 3B). As shown in Figure 4A, quercetin supplementation did not affect epididymal fat mass. The retroperitoneal fat mass was slightly lower in the quercetin-treated group than the control group but the difference was not statistically significant (*p* = 0.09, Figure 4B).

**Figure 5:**
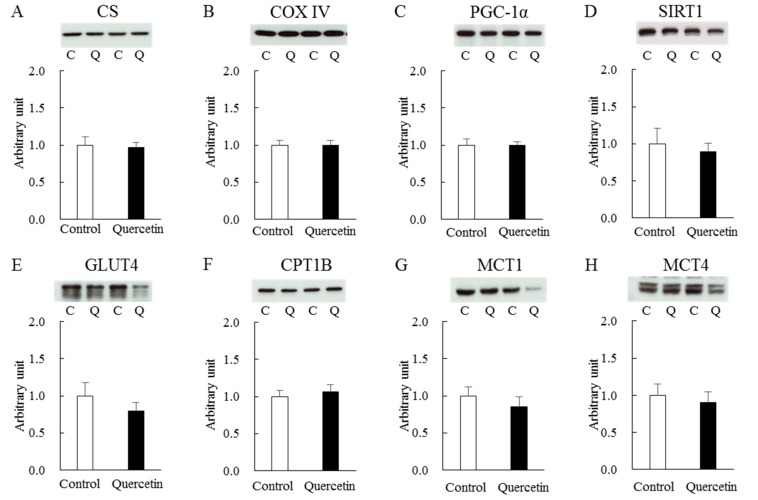
Effects of 7-day quercetin treatment on metabolic molecules in epitrochlearis muscles. Rats were assigned to either a control or quercetin groups and were maintained under ad libitum feeding for 7 days. After that, epitrochlearis muscles were dissected out as Western blot samples for CS (**A**), COX IV (**B**), PGC-1 α (**C**), SIRT1 (**D**), GLUT4 (**E**), CPT1B (**F**), MCT1 (**G**) and MCT4 (**H**). A representative blot is shown above each figures. Values are means ± SE (*n* = 6–13). All values were normalized by GAPDH abundance. C: control, Q: quercetin.

Next, we measured mitochondrial content in epitrochlearis muscle obtained at the end of the treatment period. The citrate synthase (CS) and cytochrome C oxidase (COX) IV enzymes are major indicators of mitochondrial content. Western blot analysis revealed that the levels of these proteins were not affected by quercetin treatment (Figure 5A and B). As supporting evidence, there were no effects of quercetin treatment on several proteins associated with mitochondrial biogenesis, including PGC-1α (Figure 5C) and SIRT1 (Figure 5D). To better understand the metabolic impact of quercetin on skeletal muscle, we quantified the protein expression levels of critical molecules in glycolytic/glycogenolytic and lipid metabolisms, namely glucose transporter (GLUT) 4 (Figure 5E), carnitine palmitoyltransferase (CPT) 1B (Figure 5F), monocarboxylate transporter (MCT) 1 (Figure 5G) and MCT4 (Figure 5H). The two groups did not differ in the expression levels of any of these molecules. Level of glyceraldehyde-3-phosphate dehydrogenase (GAPDH) was identical between the two groups (control, 1.00 ± 0.03; quercetin, 0.99 ± 0.04).

**Figure 6:**
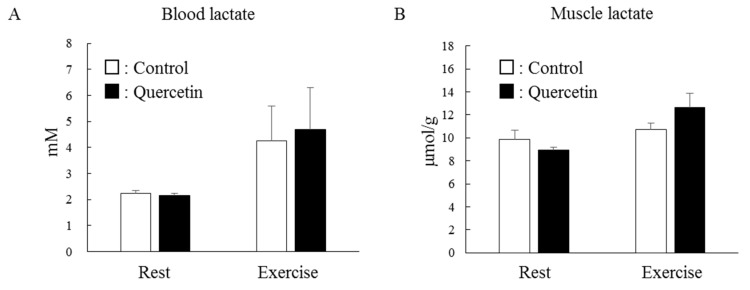
Effects of 7-day quercetin treatment on exercise-induced lactate metabolism. Rats were assigned to either a control or quercetin groups and were maintained under ad libitum feeding for 7 days. After that, rats underwent swimming exercise for 1 h. Immediately after exercise, blood was taken for blood lactate (**A**) and epitrochlearis muscles were dissected out as samples for muscle lactate (**B**). Values are means ± SE (*n* = 6–7).

We next examined whether chronic quercetin treatment affected energy metabolism during exercise. After the treatment period, the blood lactate level did not differ between the two groups at rest (basal level) (Figure 6A). Immediately after endurance exercise, the blood lactate level increased approximately two-fold but this change was identical in both groups. Similarly, the lactate level in epitrochlearis muscle was identical in both groups at rest and immediately after exercise (Figure 6B).

**Figure 7:**
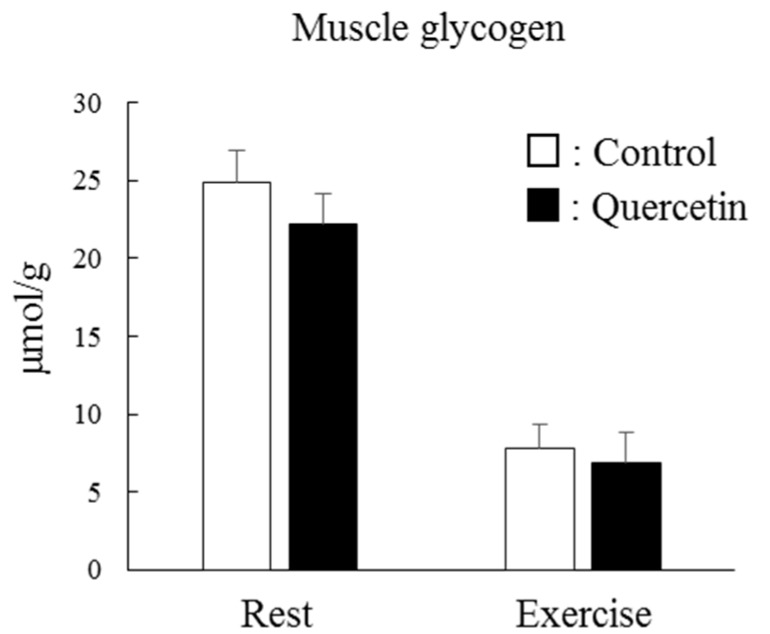
Effects of 7-day quercetin treatment on exercise-induced glycogen metabolism. Rats were assigned to either a control or quercetin groups and were maintained under ad libitum feeding for 7 days. After that, rats underwent swimming exercise for 1 h. Immediately after exercise, epitrochlearis muscles were dissected out as samples. Values are means ± SE (*n* = 6–7).

The same magnitude of the lactate levels shown in Figure 6 are associated with glycogen levels at rest and during exercise (Figure 7). At rest, the glycogen level in epitrochlearis muscle was unaffected by quercetin supplementation. One-hour endurance exercise resulted in substantial consumption of muscle glycogen in both groups but the magnitudes in the two groups were comparable. 

**Figure 8:**
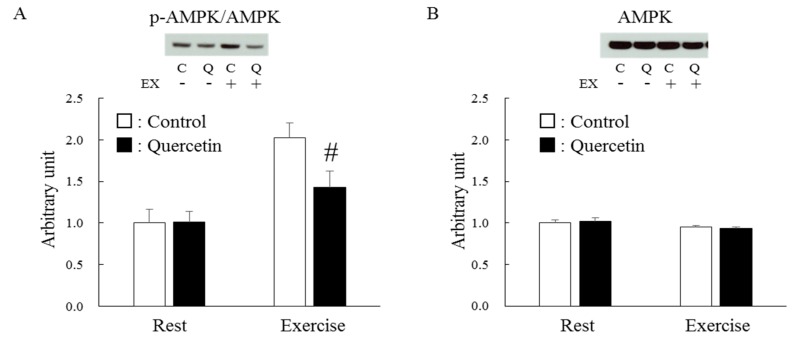
Effects of 7-day quercetin treatment on exercise-induced AMPK phosphorylation. Rats were assigned to either a control or quercetin groups and were maintained under ad libitum feeding for 7 days. After that, rats underwent swimming exercise for 1 h. Immediately after exercise, epitrochlearis muscles were dissected out as Western blot samples for p-AMPK (**A**) and AMPK (**B**). A representative blot is shown above each figures. The obtained values of p-AMPK were normalized with the value for AMPK. Values are means ± SE (*n* = 6–7). #*p* < 0.05 vs. control in exercised group. C: control, Q: quercetin, EX: exercise, EX (+): exercised, EX (-): rest.

Figure 8A shows the effect of exercise on AMPK phosphorylation levels in epitrochlearis muscle. Before exercise, quercetin had no effect. Immediately after exercise, there was a two-fold increase in the AMPK phosphorylation level in the control group and a significantly smaller increase in the quercetin group (*p* < 0.05). This reduced response to exercise was not due to a difference in AMPK content (Figure 8B). 

### 3.4. Chronic (7 Days) Effect of Quercetin Treatment (with Soleus Muscle)

**Figure 9:**
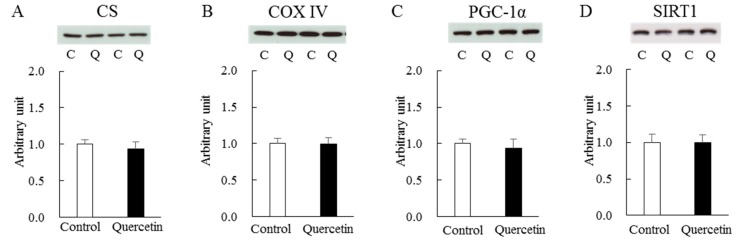
Effects of 7-day quercetin treatment on metabolic molecules in soleus muscles. Rats were assigned to either a control or quercetin groups and were maintained under ad libitum feeding for 7 days. After that, epitrochlearis muscles were dissected out as Western blot samples for CS (**A**), COX IV (**B**), PGC-1α (**C**) and SIRT1 (**D**) A representative blot is shown above each figures. Values are means ± SE (*n* = 7). All values were normalized by GAPDH abundance. C: control, Q: quercetin.

The aforementioned results were obtained using epitrochlearis muscle, which consists primarily of fast-twitch glycolytic fibers. The previous report by Davis et al. [7], showing a positive effect of quercetin on mitochondrial biogenesis, was obtained using soleus muscle, which is composed mainly of slow-twitch oxidative fibers. We therefore performed additional analyses of mitochondrial biogenesis-associated proteins in soleus muscle following 7-day quercetin supplementation (Figure 9). As was the case in the experiments using epitrochlearis muscle, the protein expression levels of CS (Figure 9A), COX IV (Figure 9B), PGC-1α (Figure 9C) and SIRT1 (Figure 9D) were not increased by quercetin treatment. Levels of p-AMPK (control, 1.00 ± 0.02; quercetin, 0.99 ± 0.04), AMPK (control, 1.00 ± 0.18; quercetin; 0.79 ± 0.11) and GAPDH (control, 1.00 ± 0.05; quercetin, 1.03 ± 0.06) were identical between the two groups.

## 4. Discussion

A previous rodent study conducted by Henagan et al. [25] demonstrated the effect of 3- and 8-week quercetin treatment on mouse muscle PGC-1α mRNA under a high-fat diet and found that low-dose treatment with 50 µg/day for 8 weeks but not 3 weeks, significantly increased PGC-1α mRNA, whereas high-dose treatment with 600 µg/day did not increase mRNA levels with either treatment duration. The study by Henagan et al. [25] did not measure the protein levels of PGC-1α or mitochondrial enzymes. More recently, the same group also reported that mice treated with low-dose quercetin for 9 weeks under a high-fat diet exhibited greater mitochondrial content in mouse muscle than control mice fed the same diet [26]. The authors assessed mitochondrial content by measuring mtDNA but the validity of mtDNA as a marker of mitochondrial content has been debated because, in some cases, mitochondrial enzyme activity and protein content do not correlate with mtDNA levels [7,27,28]. In the present study, we conducted protein-level analysis of mitochondrial enzymes (CS and COX IV). Also, we analyzed two different muscles, one rich in slow-twitch oxidative fibers (soleus) and the other composed mainly of first-twitch glycolytic fibers (epitrochlearis). This was done because these fiber types differ in several ways, for instance the relative abundance of basal mitochondrial content and SIRT1 protein [29], which could result in different capacities for mitochondrial adaptation. Our results clearly demonstrated that quercetin treatment did not stimulate mitochondrial biogenesis in skeletal muscle regardless of muscle fiber type. In the present study, we used a high-dose quercetin treatment (25 mg/kg BW/day). Although quercetin treatment might have properties related to hormesis effect in mitochondrial biogenesis, this dose used in the present study was reported to induce dramatic increase in mitochondrial proteins [7].

We cannot explain this discrepancy. However, the fact that quercetin failed to influence mitochondrial biogenesis in vivo was supported by our finding that it also did not affect SIRT1 or PGC-1α protein levels. To further characterize why quercetin did not impact skeletal muscle, we focused on AMPK, the primary molecule responsible for stimulating mitochondrial biogenesis. In previous studies, quercetin induced AMPK activation when C2C12 and L6 myotubes were directly incubated with quercetin in vitro [14,15,16]. Unlike experiments using ~100 μM resveratrol [17,24] and studies that evaluated ~100 μM quercetin treatment of cultured cells [14,15,16], we demonstrated that 100 μM quercetin did not directly stimulate AMPK activation in mature, intact skeletal muscle. Quercetin aglycone is used frequently in such experiments but this form of quercetin is known to be rare or undetectable in the blood of humans and rats [30,31,32]. In the case of oral ingestion of quercetin, several quercetin metabolites were previously shown to appear in the blood [30,31,32]. Although the blood concentrations of these metabolites and their direct action on AMPK activity in skeletal muscle were not examined in the present study, these metabolites may affect muscle AMPK directly or indirectly after oral ingestion of quercetin. To examine this possibility, we measured AMPK activation in muscles 4 h after a single (acute) oral dose of quercetin and also 16 h after the last oral ingestion during chronic treatment. Again, our results demonstrated that quercetin did not affect muscle AMPK activation after oral challenge. This is a plausible reason why quercetin treatment does not induce muscle mitochondrial biogenesis in vivo.

It is agreed that mitochondrial content is important for maximal endurance performance but it is not the sole determinant. We examined whether quercetin had ergogenic effects other than mitochondrial biogenesis. Another factor is glycogen metabolism, which is associated with the availability of lactate and free fatty acid (FFA). Lactate levels are controlled by the rates of lactate production and utilization. Lactate is an easily utilized substrate that is proactively transported during endurance exercise by MCT1 and MCT4 proteins, which are responsible for lactate uptake and extrusion, respectively, across the muscle cell membrane [33]. Given that exercise training induces MCT1 and MCT4 proteins [33], upregulation of these molecules has been implicated as a positive adaptation for enhancing endurance performance. So far, no studies have tested the effect of quercetin on these important proteins. In the present study, we showed for the first time that quercetin treatment had no impact on either MCT1 or MCT4 and that lactate metabolism during exercise, as measured by blood and muscle lactate levels, was unchanged. Since AMPK activation is thought to upregulate MCT proteins [33,34], our results regarding MCT1 and MCT4 are supported by present evidence showing that quercetin did not affect AMPK activation levels. 

The increase in blood FFA levels during endurance exercise also contributes to improved endurance capacity, specifically by coordinating the regulation of glycogen metabolism. Greater oxidation of FFAs, as observed in trained subjects, reduces glycogen use and increases endurance capacity. Maintaining higher glycogen levels before exercise also delays glycogen depletion during exercise. Only one previous study measured glycogen levels at rest and during exercise in quercetin-treated subjects. Dumke et al. demonstrated [35] that 3-week treatment with quercetin in trained athletes did not alter glycogen utilization speed during endurance exercise. Consistent with this human study, we showed that quercetin treatment of rodents did not influence glycogen levels either at rest or immediately after endurance exercise. Furthermore, the metabolic molecules GLUT4 and CPT1B, which may alter glycogen utilization speed by facilitating glucose and FFA uptake into muscle cells, were not influenced by quercetin treatment. We did not identify any adaptations in response to quercetin treatment that would exert an ergogenic effect on endurance performance.

While many studies have reported quercetin’s ergogenic effect on endurance exercise [6,7,8,9], other studies have shown no effect of chronic quercetin treatment [11,36,37]. Importantly, Casuso et al. [27] demonstrated that chronic quercetin treatment and exercise training impaired training-induced citrate synthase adaptation in skeletal muscle. This detrimental effect of quercetin on mitochondria might mask some of quercetin’s ergogenic effects, especially when quercetin is administered to subjects engaged in exercise training. In the present study, quercetin treatment blunted exercise-induced AMPK activation. Although precise role of AMPK activation in exercise-induced muscle adaptation is still under debate [20], this might contribute to the decreased training-induced mitochondrial adaptation [27]. Identifying the mechanisms underlying the reduced AMPK response to exercise is beyond the scope of this study; however, given that production of reactive oxygen species and reactive nitrogen species are increased during muscle contractile activity and that these small molecules are capable to stimulate AMPK activation [18,19], cancellation of these molecules during exercise by quercetin’s antioxidant function might play a role in this phenomenon. 

In contrast, the present study showed that the level of glycogen, a negative AMPK regulator, apparently did not contribute to the reduced AMPK response because the glycogen level was the same at baseline and immediately after exercise. We examined the exercise-induced AMPK activation level only in the epitrochlearis muscle, located in the upper arm, because this muscle is a predominant working muscle in the swimming exercise model [38]. As a result, we could not extrapolate the evidence obtained in this glycolytic muscle to oxidative muscles such as the soleus. However, our results indicate that repetitive quercetin treatment might worsen AMPK-induced muscle adaptation, including mitochondrial biogenesis.

As experimental limitations in this study, we cannot mention more about quercetin’s hormesis effect and ineffectual term because we did experiments with one dose and one term for the treatment. The longer-term effect of quercetin treatment needs to be examined in the future. Also, we could not examine whether the blunted exercise-induced AMPK activation actually might result in any detrimental effect in exercise training.

## 5. Conclusions

The following results of this study are of special interest to athletes: 1) quercetin treatment did not stimulate mitochondrial biogenesis in skeletal muscle, 2) quercetin treatment did not influence metabolism in a way that might enhance endurance exercise capacity and 3) quercetin treatment might provide a disadvantage to muscle adaptation when administered with exercise training. The molecular results of this study indicate that quercetin treatment may not be advantageous for improving endurance exercise performance, at least after high-dose and short-term therapy.
